# Simultaneous Determination of 13 Constituents of Radix Polygoni Multiflori in Rat Plasma and Its Application in a Pharmacokinetic Study

**DOI:** 10.1155/2020/4508374

**Published:** 2020-03-03

**Authors:** Wenhao Cheng, Yinghui Li, Wei Yang, Siyang Wu, Mengmeng Wei, Yang Gao, Chen Kang, Shuofeng Zhang, Yingfei Li

**Affiliations:** ^1^School of Chinese Pharmacy, Beijing University of Chinese Medicine, Beijing 100102, China; ^2^Center for DMPK Research of Herbal Medicines, Institute of Chinese Materia Medica, China Academy of Chinese, Beijing 100700, China

## Abstract

Radix Polygoni Multiflori (RPM) has been widely used to treat various diseases in Asian countries for many centuries. Although, stilbenes and anthraquinones, two major components of RPM, show various bioactive effects, it has been speculated that the idiosyncratic hepatotoxicity induced by RPM may be related to these constituents. However, information on the pharmacokinetics of stilbenes and anthraquinones at a subtoxic dose of RPM is limited. A simple and sensitive UPLC-MS/MS bioanalytical method for the simultaneous determination of 13 ingredients of RPM, including chrysophanol, emodin, aloe-emodin, rhein, physcion, questin, citreorosein, questinol, 2,3,5,4′-tetrahydroxystilbene-2-O-*β*-D-glucoside, torachrysone-8-O-glucoside, chrysophanol-8-O-*β*-D-glucoside, emodin-8-O-*β*-D-glucoside, and physcion-8-O-*β*-D-glucoside, in rat plasma was established. Acetonitrile was employed to precipitate the plasma with appropriate sensitivity and acceptable matrix effects. Chromatographic separation was performed using a waters HSS C18 column with a gradient elution using water and acetonitrile both containing 0.025% formic acid within a run time of 9 min. The constituents were detected in negative ionization mode using multiple reaction monitoring. The method was fully validated in terms of selectivity, linearity, accuracy, precision, recovery, matrix effects, and stability. The lower limit of quantitation of the analytes was 0.1–1 ng/mL. The intrabatch and interbatch accuracies were 87.1–109%, and the precision was within the acceptable limits. The method was applied to a pharmacokinetic study after oral administration of RPM extract to rats at a subtoxic dose of 36 g/kg.

## 1. Introduction

Radix Polygoni Multiflori (RPM, Heshouwu in Chinese), the tuberous roots of *Polygonum multiflorum* Thunb. (Polygonaceae), is one of the most popular traditional Chinese medicines (TCMs) and has been used to treat hyperlipidemia, coronary heart disease, neurosis, and other diseases commonly associated with aging in China and other Asian countries for many centuries [1–3]. Besides its medical uses, RPM has been made as tonic food and beverages and has become popular as a result of the growing interests of general population in phytonutrients and alternative medicines during the past decades. Although it is officially documented in the Chinese Pharmacopoeia, the safety profile of PMR has attracted wide concern due to recently increased reports of hepatic impairment resulting from the use of RPM and RPM-containing herbal products. Accordingly, the recommended PMR dose in the Chinese Pharmacopoeia was adjusted from 6 to 12 g in the 2005 edition to 3–6 g in the 2010 edition due to safety concerns [4, 5]. The potential liver toxicity of PMR in rats is significant with increasing dose to 20 g crude drug/kg (60-fold clinical dose) [6]; however, hepatotoxicity associated with RPM is also idiosyncratic [7] and not related to the dose, route, or duration of drug administration [8].

Unlike Western medicine, TCMs are complex chemical mixtures. The effect of an herbal therapy is not necessarily the result of a single mechanism induced by a single ingredient but a range of activities of multiple compounds working together to produce a medicinal benefit. Although more than 140 compounds were detected in PMR extracts [9], stilbenes and anthraquinones are two major characteristic constituents of RPM. Stilbenes, mainly 2,3,5,4′-tetrahydroxystilbene-2-O-*β*-D-glucoside (TSG), possessed antioxidative, antitumor, anti-inflammatory, endothelial protective, neuroprotective, and liver-protective activities, etc. [10–21]. Anthraquinones were reported to possess many biological activities, including immunomodulating, anticancer, antimutation, antibacterial, anticancer, antioxidant, etc. [22–25], with a different effects between free and glycoside forms [26]. However, the hepatotoxic chemicals attributed to RPM-induced idiosyncratic hepatotoxicity [27] remain in dispute [28]. It has been speculated that the toxicity may be related to stilbenes [29] or anthraquinones [30, 31]. One of the factors that determine susceptibility to uncommon idiosyncratic reactions is that the unique disposition of the drug metabolism manipulating under genetic polymorphisms can alter exposure to toxic metabolites and exceeding the threshold [32]. For example, TSG was able to accelerate the exposure and metabolism of emodin to increase potential PMR-induced liver injury through upregulation activity of CYP1A2 isozyme [33]. Hence that, evaluating the pharmacokinetic behavior of the active constituents of RPM can offer us valuable information for better understanding their pharmacological and/or toxicological effects.

There were articles that had described the pharmacokinetic properties of constituents of RPM, mainly focused on few constituents in RPM [31, 34–36]. In fact, studies of those individual components and/or with single administration of PMR constituents do not reflect the real pharmacokinetic characteristics after administration of RPM. Therefore, it is necessary and meaningful to develop an accurate and selective bioanalytical method for the simultaneous determination of more biological ingredients in plasma to understand the characterization and diversity of the pharmacokinetic properties of RPM. The ultraperformance liquid chromatography combined with triple quadrupole tandem mass spectrometry (UPLC-MS/MS), detection using different multiple reaction monitor (MRM) channels at the same time, is a powerful technique used for simultaneous quantification of multiple components in complex matrix due to its very high sensitivity and selectivity. Several LC-MS/MS analytical methods determining multicomponents of RPM and the application of pharmacokinetics-related investigations were reported [31, 33, 37, 38]. However, at most 7 analytes of RPM were simultaneous determined according to the reports [38]. Therefore, the aim of this study was to develop and validate a method for simultaneous measurement of 13 components (chrysophanol, emodin, aloe-emodin, rhein, physcion, questin, citreorosein, questinol, TSG, torachrysone-8-O-glucoside (TG), chrysophanol-8-O-*β*-D-glucoside (CG), emodin-8-O-*β*-D-glucoside (EG), and physcion-8-O-*β*-D-glucoside (PG)) in rat plasma using an UPLC-MS/MS and investigate their pharmacokinetics after rats administration of RPM extract.

## 2. Materials and Methods

### 2.1. Chemicals and Materials

**Figure 1:**
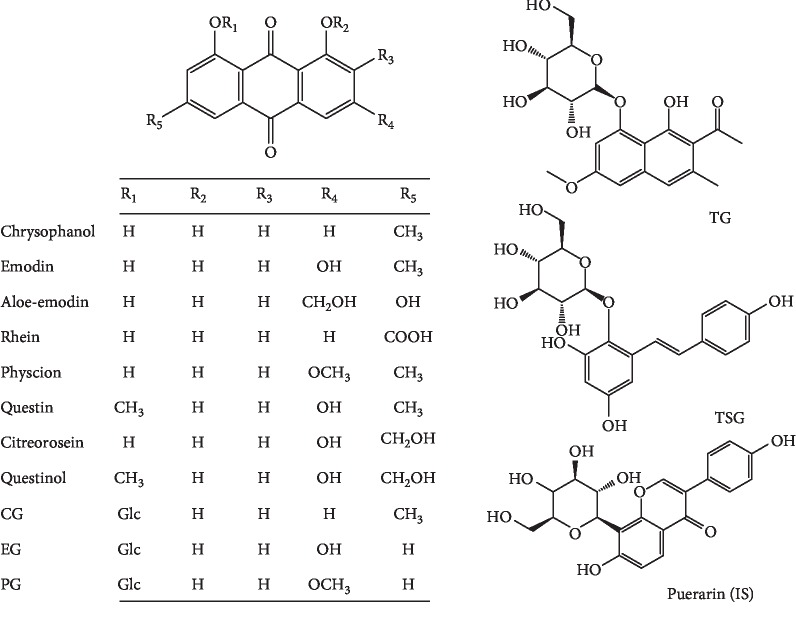
Chemical structures of the 13 constituents of RPM and the IS.

Standards of chrysophanol, emodin, aloe-emodin, rhein, physcion, questin, TSG, emodin-8-O-*β*-D-glucoside (EG), physcion-8-O-*β*-D-glucoside (PG), and puerarin (IS) were purchased from the Chengdu Chroma-Biotechnology Co., Ltd. (Chengdu, China). Citreorosein, questinol, torachrysone-8-O-glucoside (TG), and chrysophanol-8-O-*β*-D-glucoside (CG) were purchased from the Qingdao Advancechem Technology Co., Ltd. (Qingdao, China). The chemical structures of the 13 constituents of RPM and IS are shown in Figure 1.

RPM was purchased from Beijing Tongrentang Pharmaceutical Co., Ltd, and identified by professor Shuofeng Zhang (Beijing University of Chinese Medicine). The voucher specimen (lot no.: YDZY-HSW-20180521) was deposited at 4°C in our laboratory. The RPM (200 g) was refluxed with 70% ethanol twice (1 : 10, w/v), 3 h for each time. All the solution obtained were put together and condensed by vacuum drier at 50°C to get 50 ml extract, containing 4 g raw herb per milliliter.

LC-grade acetonitrile and formic acid were purchased from Honeywell (Morristown, USA) and Roe Scientific Inc. (Newark, USA), respectively. Ultra-pure water was prepared using a Millipore Milli-Q purification system (Bedford, USA).

### 2.2. LC-MS/MS Instrument and Analytical Conditions

The analysis was performed on an AB Sciex API 5500 *Q* Trap mass spectrometer (Toronto, Canada), interfaced with a Waters Acquity UPLC separation module. Empower 3.0 and Analyst 1.62 software were used to control UPLC and mass spectrometer, respectively.

Chromatographic separation was achieved on a waters HSS C18 column (100 × 2.1 mm, 1.8 *μ*m, kept at 40°C) using a mobile phase containing 0.025% formic acid that consisted of solvent A (water) and solvent B (acetonitrile). The mobile phase was delivered at 0.3 mL/min, and a gradient program was used as follows: 0-1 min, held 5% to 25% solvent B; 1–3.5 min, linear gradient from 25% to 35% solvent B; 3.5–5 min, linear gradient from 35% to 40% solvent B; 5-6 min, linear gradient from 40% to 60% solvent B; 6-6.1 min, linear gradient from 60% to 95% solvent B; 6.1–8 min, linear gradient from 95% to 100% solvent B; and 8-9 min, held 5% solvent B.

The MS detection was set in positive multiple reaction monitoring (MRM) mode for the analytes and IS. The turbo spray temperature was maintained at 500°C. The nebulizer gas (gas 1), heater gas (gas 2), and curtain gas was set at 45, 50, and 45 psi, respectively. The interface heater was on. The precursor ion, corresponding product ion and dwell time along with declustering potential (DP), entrance potential (EP), collision energy (CE), and collision exit potential (CXP) for each compound were optimized with standard substance. The dwell time was 50 ms for all analytes. The EP and CXP were set at –10 V and −16 V, respectively. The other detailed mass spectrometric conditions are shown in Table 1.

### 2.3. Working Solutions and Quality Control (QC) Samples

The stock of standard solution was prepared as chrysophanol, emodin, aloe-emodin, rhein, physcion, questin, citreorosein, questinol, TSG, TG, CG, EG, PG, and IS at a concentration of 1 mg/mL. The calibration samples of analytes were prepared by adding a series of different concentration working solution (5 *μ*L) to drug-free rat plasma (45 *μ*L). Quality control (QC) samples were prepared independently at the concentrations shown in Table 2 for the method validation. All stock solutions and working solutions were stored at −70°C pending use.

### 2.4. Sample Preparations

Sample preparations were made using the protein precipitation process. 50 *μ*L thawed plasma, 10 *μ*L IS working solution (puerarin 10 *μ*g/mL), and 150 *μ*L acetonitrile were added to a 1.5 mL tube in that order. After vortexed for 5 min and centrifuged at 12,000 g for 5 min at 4°C, 150 *μ*L of the supernatant was transferred and a 5 *μ*L was injected into the UPLC-MS/MS instrument for analysis.

### 2.5. Method Validations

The developed LC-MS/MS method was performed through complete method validation based on the industrial guidelines for bioanalytical method validation from the US FDA [39].

#### 2.5.1. Specificity, Selectivity, LLOQ, and Linearity

The blank rat plasma samples from six sources, blank plasma samples spiked with analytes at the lower limit of quantification (LLOQ), and plasma samples obtained from PK studies were analyzed to ascertain the specificity and selectivity of the method for endogenous plasma matrix components.

The LLOQ for the analytes in rat plasma were defined as the lowest concentration giving a signal-to-noise ratio of at least 10, acceptable accuracies of 80–120%, and sufficient precisions within 20%. These were verified by 5 replicate analyses.

Matrix-matched calibration curves were generated by plotting the peak ratios of the analytes to IS vs the nominal concentrations of the calibration standards at 0.1, 0.3, 0.9, 2.7, 8.1, 24.3, and 72.9 ng/mL for emodin, aloe-emodin, questin, citreorosein, questinol, TG, CG, EG, PG; 0.3, 0.9, 2.7, 8.1, 24.3, 72.9, and 218.7 ng/mL for TSG; 0.5, 1.5, 4.5, 13.5, 40.5, 121.5, and 364.5 ng/mL for rhein and physcion; and 1, 3, 9, 27, 81, 243, and 729 ng/mL for chrysophanol. Matrix-matched calibration curves were constructed using weighted (1/*X*^2^) linear regression of the peak area of compounds to internal standard (*Y*) against the corresponding nominal compound concentration (*X*, ng/mL). All the values, except LLOQ, were accepted within 15% of nominal concentration.

#### 2.5.2. Accuracy and Precision

To assess the intraday and interday precision and accuracy, complete analytical runs were performed on the same day and on three different days, respectively. Intraday precision and accuracy were determined by analyzing six replicates of the LLOQ samples and three different QC samples on the same day. Interday precision and accuracy were also evaluated by analyzing 18 replicates of the LLOQ sample and three different QC samples on three different days (six replicates/day). Precision was expressed as the relative standard deviation (RSD, %), and the accuracy was expressed as follows: (observed concentration/nominal concentration) × 100%.

#### 2.5.3. Recovery and Matrix Effect

The recoveries and matrix effects of the analytes were evaluated to investigate the efficiency of the assay process using three sets samples. Recovery was calculated comparing the peak area of the analytes spiked before extraction (Set 3) with the mean peak area of analytes spiked postextraction (Set 2) in three QC concentrations. Matrix effects were determined at the same three QC concentrations and were calculated as the area ratio of the analytes spiked postextraction (Set 2) to the mean same analytes area in neat standard solution (Set 1). The matrix effect were calculated by (area of analytes in Set  2/Mean  area  of analytes  in  of  Set  1) × 100% and CV of (area of  analytes  in  Set   2/Mean area of analytes in of Set  1) × 100% as absolute matrix effect and relative matrix effect, respectively. The relative matrix effect should not be greater than 15%. The recovery was calculated as (area of analytes in Set  3/mean area of analytes in Set  2) × 100%.

#### 2.5.4. Stability

The stability of the 13 analytes in rat plasma were assessed by analyzing samples spiked 0.2, 2.7, and 58.3 ng/mL for emodin, aloe-emodin, questin, citreorosein, questinol, TG, CG, EG, PG; 0.6, 8.1, and 175 ng/mL for TSG; 1, 13.5, and 291.6 ng/mL for rhein, physcion; and 2, 27, and 583.2 ng/mL for chrysophanol, respectively, under four conditions: (1) short-term storage for 4 h at room temperature; (2) long-term storage for (3 months at −70°C; (3) three freeze-thaw cycles; and (4) posttreatment storage for 24 h at 6°C setting in UPLC autosampler. The concentrations obtained were compared with the nominal values of the spiked samples. The stability was calculated as the percent of the measured concentration to the initial concentration at time zero. The analytes were considered stable if the assay values were within the acceptable limit of accuracy (85.0∼115%).

### 2.6. Application of the Method to a Pharmacokinetic Study

Male Sprague-Dawley rats (200–240 g) were obtained from Beijing Vital River Laboratory Animal Technology Co., Ltd. (Beijing, China) (SCXK 2016(jing)-0006). The animals were maintained in SPF animal room at temperature 22 ± 2°C, 60 ± 5% humidity, and 12/12 h day/night cycle. All procedures were approved by the Institutional Animal Ethics Committee and performed in accordance with the Regulations of Experiment Animal Administration issued by the State Committee of Science and Technology of China. RPM extract suspended in an equal volume of 5% CMC-Na solution and orally administered to the six rats at a single dose of 36 g/kg (dose calculated as grams of crude materials used to create the extract per kilogram of rat body weight). The dose was set at a subtoxic level [40,41] to investigate the pharmacokinetic profiles of the constituents of RPM and were approximately 72 times the upper dose (6 g/day) of human recommended by the 2015 edition of Chinese Pharmacopoeia [5] (converted to rat dose based on body surface area conversion [42]). The calculated doses of compounds based on the contents in the extract were 7.38, 14.8, 13.7, 8.82, 15.5, 11.2, 55.8, 1.08, 1170, 11.3, 25.7, 131.4, and 13.0 mg/kg for chrysophanol, emodin, aloe-emodin, rhein, physcion, questin, citreorosein, questinol, TSG, TG, CG, EG, and PG, respectively. Rats were fasted for at least 12 h before the experiments and had free access to water. Blood samples (∼0.25 mL) were collected from the retinal venous plexus into heparinized 1.5 mL polythene tubes before administration and 5, 15, and 30 min and 1, 2, 4, 6, 8, 12, and 24 h after dosing RPM extract at dose of 36 g/kg. The plasma was immediately separated from blood samples and stored frozen at −70°C until analysis.

### 2.7. PK Parameters and Statistical Analysis

The pharmacokinetic parameters were calculated using the pharmacokinetic software Phoenix® WinNonlin®, version 7.0 (Scientific Consulting Inc., Apex, NC, USA). The peak plasma concentration (*C*_max_) and time to reach *C*_max_ (*T*_max_) were read directly from the experimental data. The total area under the plasma concentration-time curve from time zero to infinity (AUC_0–∞_) or the last measured time (AUC_0–*t*_), and terminal half-life (*t*_1/2_) were estimated using noncompartmental analysis. All values were expressed as the mean ± standard deviation (SD).

## 3. Results and Discussion

### 3.1. LC-MS/MS Optimization Conditions

An UPLC-MS/MS method for the 13 analytes and IS in rat plasma was investigated. Standard solutions at 100 ng/mL were analyzed to optimize the mass spectrometry conditions. Full scans in positive and negative modes were investigated by monitoring both precursor and product ions in multiple reaction monitoring (MRM) mode to identify the maximum response of the analytes. Results showed using negative scan mode could offer higher sensitivity for the analytes. The optimized MRM parameters are listed in Table 1.

The optimization of the chromatographic condition to separate the analytes was conducted with respect to mobile phase composition, column, and elution based on sensitivity, speed, and peak shape. The use of acetonitrile with a waters HSS C18 column as the optimal mobile, respectively, to achieve good sensitivity and a better peak shape was observed. As a consequence, 0.025% formic acid solution (gradient elution) was used for high-sample throughput.

### 3.2. Method Validation

#### 3.2.1. Specificity and Selectivity

**Figure 2:**
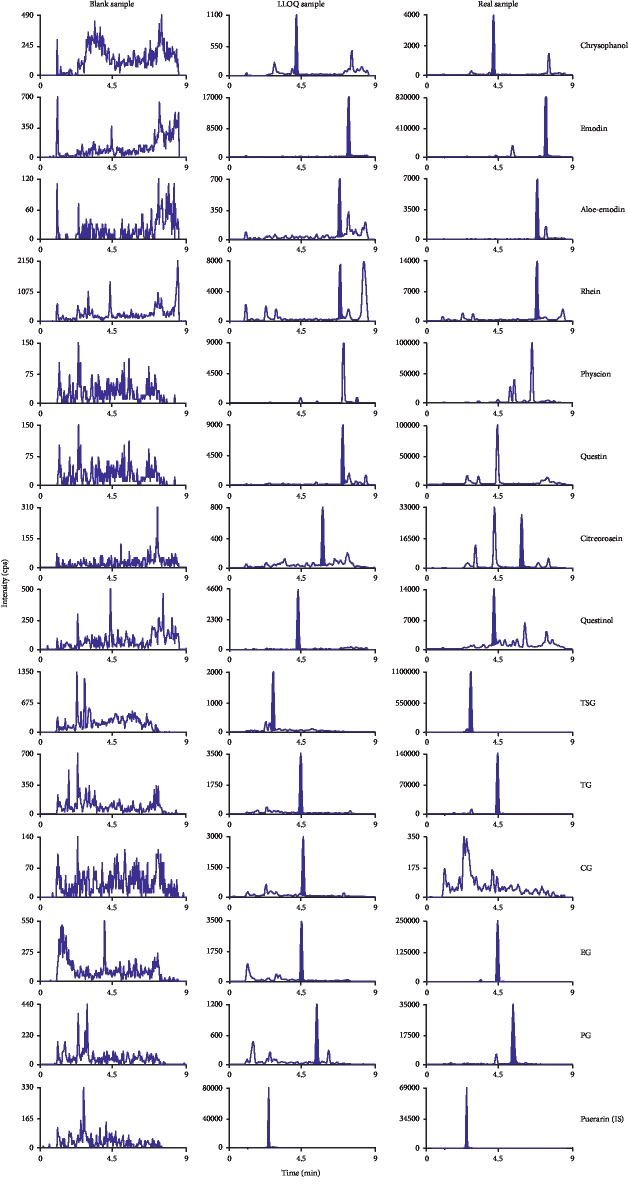
Representative MRM chromatograms of the 13 analytes and IS in blank plasma, LLOQ, and real samples. Physcion, questin, and CG were not detected in real samples.

A sensitive and reliable analytical method was developed and validated under the optimized UPLC-MS/MS conditions to investigate the pharmacokinetics of chrysophanol, emodin, aloe-emodin, rhein, physcion, questin, citreorosein, questinol, TSG, TG, CG, EG, and PG in rats. Blank plasma samples using a protein precipitation procedure have a suitable recovery with the UPLC-MS/MS conditions to ensure less interference of the analytes and internal standard (IS) from plasma. The representative chromatograms for standards of the analytes spiked in blank rat plasma, plasma containing these analytes at LLOQ concentration, and real samples collected at 60 min after administration of RPM extract are shown in Figure 2. The results show no significant interference from endogenous substances observed under the current analytical conditions, which indicated the specificity and selectivity of the elaborated procedures.

#### 3.2.2. Linearity and the Limits of Detection and Quantification

The linearity of the calibration curves was determined and analyzed with six replicates of concentrations ranging from 0.1 to 364.5 ng/mL in blank plasma samples. A calibration curve was constructed using 1/*X*^2^ as weighting factor for each concentration by comparing the peak area ratio with the internal standard. The results demonstrated linearity of 0.1–72.9 ng/mL for emodin, aloe-emodin, questin, citreorosein, questinol, TG, CG, EG, and PG, 1–729 ng/mL for chrysophanol, 0.5–364.5 ng/mL for rhein, physcion, and 0.3–218.7 ng/mL for TSG in rat plasma with correlation coefficients (*r*) > 0.99 obtained for the regression lines. The limits of detection (LOD) and quantification (LLOQ) for all 13 bioactive components were 0.1–1 ng/mL, which showed excellent reproducibility and sufficient concentrations for oral administration of the herbal formulation in the subsequent PK study (Table 2).

#### 3.2.3. Precision and Accuracy

The intraday and interday accuracy and precision (% RSD) data for the 13 components are presented in [Supplementary-material supplementary-material-1]. The intraday and interday accuracy values for the 13 analytes ranged 89.0–109% and 87.1–107%, respectively. The intraday and interday precision values for the 13 analytes ranged 2.09–10.9% and 1.83–10.3%, respectively.

#### 3.2.4. Recovery and Matrix Effect

The recovery of the 13 analytes from rat plasma was performed after the extraction procedure was assessed in three QC level samples. The mean recovery for all analytes was between 89.9% and 107% ([Supplementary-material supplementary-material-1]). The absolute matrix effects of chrysophanol, emodin, aloe-emodin, rhein, physcion, questin, citreorosein, questinol, TG, and EG were between 88.0% and 109%. However, the absolute matrix effects of TSG, CG, and PG ranged from 55.6 to 79.5%. However, the relative matrix effect was ≤13.1% for all analytes, indicating that the existed response suppression of TSG, CG, and PG comprised TSG, CG, and PG analytical sensitivity without sacrificing the accuracy and reliability ([Supplementary-material supplementary-material-1]).

#### 3.2.5. Stability

Stock solutions of the 13 analytes and the IS in methanol were stable for at least 4 weeks at −70°C. The 13 analytes were all stable in situations mimicking those encountered during sample storage, handling, and analysis, for all the test, rat plasma samples (freeze/thaw stability during and after three cycles from −70°C to room temperature, short-term stability at room temperature for 4 h, and long-term storage at −70°C for 3 months) and the supernatants resulting from the acetonitrile-precipitated plasma samples (autosampler storage stability at 6°C for 24 h) were between 87.6% and 112% of nominal concentration and well within the limits of acceptability (not exceeding ±15%, [Supplementary-material supplementary-material-1]).

### 3.3. Application to Pharmacokinetic Study

The method was acceptably validated and used to determine 13 constituents of RPM in rat plasma after orally administration of RPM extract at doses of 36 g crude herb/kg.

After oral administration of RPM extract, three constituents of RPM, including physcion, questin, and CG, were too low to be detected and could not get pharmacokinetic parameters. However, chrysophanol, aloe-emodin, and rhein were detected no longer than 6, 12, and 2 h plasma samples, respectively, after administration of RPM extract to rats.

**Figure 3:**
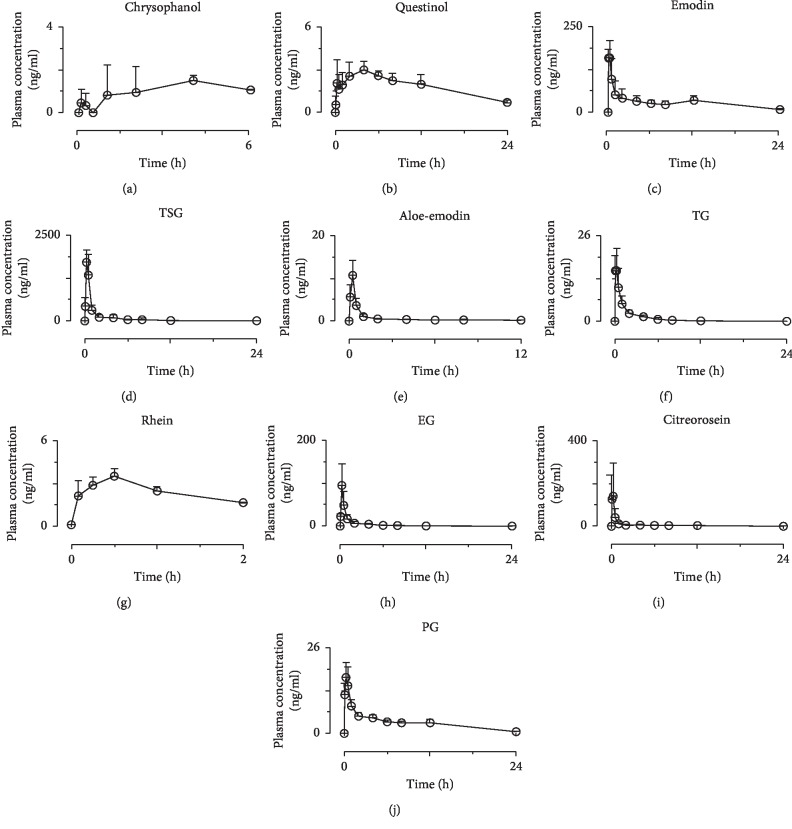
Mean plasma concentration-time profiles of the 10 constituents of RPM. The upper error bars represent the standard deviation obtained from six replicates.

The mean plasma concentration-time profiles of the detected 10 components after a single dose of RPM extract in rats are shown in Figure 3; the plasma pharmacokinetic parameters of the constituents was different as summarized in Table 3. The *T*_max_ values of emodin, aloe-emodin, rhein, citreorosein, TSG, TG, EG, and PG were 0.19 ± 0.09, 0.22 ± 0.07, 0.46 ± 0.10, 0.19 ± 0.09, 0.33 ± 0.13, 0.11 ± 0.07, 0.29 ± 0.10, and 0.26 ± 0.13 h, respectively, after single dose oral administration of RPM extract, which indicated the absorbance velocity of these compounds was relatively rapid. However, chrysophanol and questinol reached the *T*_max_ at 2.22 ± 1.76 and 4.38 ± 4.03 h, respectively, indicating a slow absorbance.

TSG reached the highest *C*_max_ (1743 ± 401 ng/mL) among the 10 constituents due to its high content in RPM extract (65.0 mg/mL). The *C*_max_ values of three constituents were higher than 100 ng/mL, including emodin (175 ± 33.8 ng/mL), citreorosein (149 ± 147 ng/mL), and EG (101 ± 47.4 ng/mL) with their contents 14.8, 55.8, and 131.4 mg/mL in RPM extract. The *C*_max_ of all other compounds ranged from 1.06 ± 0.24 ng/mL to 18.8 ± 4.85 ng/mL. Moreover, the AUC_0–*t*_, another PK parameter reflecting the levels of systemic exposure, of TSG was 1871 ± 554 ng·h/mL. However, the dose of citreorosein (55.8 mg/kg) in RPM extract was about 3.8-fold that of emodin (14.8 mg/kg) and the AUC_0–*t*_ values of citreorosein (134 ± 96.4 ng·h/mL) was 6.0-fold that of emodin (801 ± 187 ng·h/mL). The relative bioavailability of citreorosein, calculating using the AUC_0–*t*_ normalized by molecular weight and dose, was 15.8% of that of emodin due to the one more hydroxyl in the structure of citreorosein (1,3,8-trihydroxy-6-(hydroxymethyl)anthracene-9,10-dione) comparing with the structure of emodin (1,3,8-trihydroxy-6-methylanthracene-9,10-dione) (Figure 1). The AUC_0–*t*_ values of all other compounds ranged from 4.95 ± 1.90 ng·h/mL to 84.1 ± 8.95 ng·h/mL. The different content of 10 compounds in RPM extract was one of the reasons leading to different systemic exposure. In addition, as report goes, many natural compounds obtained from herb materials have been identified as substrates, inhibitors, or inducers of various CYPs, and the abovementioned values illustrated that it was possible to have impact on the system exposure of some compounds.

Moreover, the *t*_1/2_ of chrysophanol, emodin, aloe-emodin, rhein, citreorosein, questinol, TSG, TG, EG, and PG was 3.18 ± 0.62, 8.37 ± 4.17, 3.44 ± 1.40, 1.18 ± 0.39, 3.97 ± 1.31, 8.90 ± 2.70, 5.98 ± 2.62, 2.00 ± 0.63, 3.92 ± 2.50, and 6.13 ± 1.06 h, respectively. The slow elimination, including compounds of chrysophanol, emodin, aloe-emodin, citreorosein, questinol, TSG, EG, and PG, may be attributed to the complexity of Chinese medicine composition.

## 4. Conclusions

A simple, sensitive, and reliable UPLC-MS/MS method for the determination of the glycosides and aglycones of anthraquinones and 2,3,5,4′-tetrahydroxystilbene-2-O-*β*-D-glucoside in rat plasma was developed. This method is faster and more high-throughput with analytical time shortening from 18 min to 9 min while the number of simultaneous determined analytes increasing from 7 to 13 comparing with the previous reported method [38]. The method was acceptably validated and applied to a pharmacokinetic study of the constituents after oral administration of RPM extract in rats. The absorption of the glycosides of anthraquinones in an intact form was confirmed in the pharmacokinetic study.

The study of RPM should involve elucidating the PK characteristics of the multiple herbal compounds from RPM and understanding their fates in the body. The results of this study could be relevant to a better understanding of the pharmacokinetics and pharmacodynamics of anthraquinone glycosides and aglycones. These results demonstrated the pharmacokinetics of active ingredients of RPM in vivo and provided useful information for further bridge the gap between the complex chemical composition of the RPM and its pharmacological and/or toxicological effects.

## Figures and Tables

**Table 1:** The optimized mass spectrometry parameters of the 13 constituents of RPM and IS.

Compound	Q1 mass (Da)	Q3 mass (Da)	DP (V)	CE (V)
Chrysophanol	253.0	225.1	−98	−37
Emodin	269.0	241.0	−60	−36
Aloe-emodin	269.1	240.1	−100	−31
Rhein	283.0	256.1	−60	−35
Physcion	283.3	240.1	−60	−35
Questin	283.1	240.1	−80	−36
Citreorosein	285.0	211.0	−60	−43
Questinol	299.0	256.1	−60	−35
TSG	405.0	311.0	−80	−25
TG	407.0	245.0	−60	−23
CG	415.0	253.0	−60	−20
EG	431.1	269.0	−120	−120
PG	445.0	283.0	−80	−17
Puerarin (IS)	415.0	295.0	−200	−40

**Table 2:** Calibration curves, correlation coefficients, linear ranges, and LLOQ of the 13 analytes.

Compound	Regression equation	Linearity (*r*)	Linear range (ng/mL)
Chrysophanol	*Y* = 0.0828*X* + 0.00155	0.9995	1–729
Emodin	*Y* = 0.907*X* + 0.0512	0.9993	0.1–72.9
Aloe-emodin	*Y* = 0.0652*X* + 0.00257	0.9994	0.1–72.9
Rhein	*Y* = 0.465*X* + 0.119	0.9965	0.5–364.5
Physcion	*Y* = 0.0253*X* + 0.0016	0.9948	0.5–364.5
Questin	*Y* = 1.14*X* + 0.00681	0.9967	0.1–72.9
Citreorosein	*Y* = 0.102*X* + 0.00161	0.9998	0.1–72.9
Questinol	*Y* = 0.636*X* + 0.00179	0.9996	0.1–72.9
TSG	*Y* = 0.1*X* + 0.0259	0.9969	0.3–218.7
TG	*Y* = 0.592*X* + 0.00443	0.9993	0.1–72.9
CG	*Y* = 0.138*X* + 0.00767	0.9995	0.1–72.9
EG	*Y* = 0.54*X* + 0.0193	0.9991	0.1–72.9
PG	*Y* = 0.0576*X* + 0.00158	0.9995	0.1–72.9

**Table 3:** Pharmacokinetic parameters of 10 analytes after oral administration of RPM extract (*n* = 6).

Compound	*C*_max_ (ng/ml)	*T*_max_ (h)	*t*_1/2_ (h)	AUC_0–*t*_ (ng·h/ml)	AUC_0–∞_ (ng·h/ml)	MRT_0–∞_ (h)
Chrysophanol	1.90 ± 0.56	2.22 ± 1.76	3.18 ± 0.62	4.28 ± 1.64	10.3 ± 1.87	6.76 ± 2.06
Emodin	175 ± 33.8	0.19 ± 0.09	8.37 ± 4.17	686 ± 187	801 ± 233	11.8 ± 5.27
Aloe-emodin	11.3 ± 3.10	0.22 ± 0.07	3.44 ± 1.40	7.28 ± 3.25	8.46 ± 3.44	3.17 ± 1.54
Rhein	1.06 ± 0.24	0.46 ± 0.10	1.18 ± 0.39	1.11 ± 0.63	2.31 ± 0.51	1.83 ± 0.56
Citreorosein	149 ± 147	0.19 ± 0.09	3.97 ± 1.31	131 ± 96.9	134 ± 96.4	5.32 ± 1.85
Questinol	1.20 ± 0.20	4.38 ± 4.03	8.90 ± 2.70	14.2 ± 2.06	17.3 ± 2.84	13.6 ± 4.21
TSG	1743 ± 401	0.33 ± 0.13	5.98 ± 2.62	1811 ± 554	1871 ± 581	3.66 ± 0.97
TG	18.7 ± 4.06	0.11 ± 0.07	2.00 ± 0.63	21.2 ± 7.38	21.7 ± 7.48	2.10 ± 0.24
EG	101 ± 47.4	0.29 ± 0.10	3.92 ± 2.50	82.5 ± 32.1	83.7 ± 32.3	2.88 ± 1.41
PG	18.8 ± 4.85	0.26 ± 0.13	6.13 ± 1.06	79.5 ± 8.95	84.1 ± 8.31	8.58 ± 1.72

## Data Availability

The data used to support the findings of this study are available from the corresponding author upon request.

## Supplementary Materials

Supplementary Materials Table S1: intraday and interday accuracy and precision for the determination of the 13 constituents of RPM in rat plasma. Table S2: recovery and matrix effect of the 13 constituents of RPM in rat plasma. Table S3: stabilities of the 13 constituents of RPM in rat plasma.
