# Supplementary material for: Simultaneous Determination of 13 Constituents of Radix Polygoni Multiflori in Rat Plasma and Its Application in a Pharmacokinetic Study
Source: Int J Anal Chem. 2020 Mar 3;2020:4508374. doi: 10.1155/2020/4508374 (PMC7072103; doi:10.1155/2020/4508374)
Supplement: Supplementary Materials — Table S1: intraday and interday accuracy and precision for the determination of the 13 constituents of RPM in rat plasma. Table S2: recovery and matrix effect of the 13 constituents of RPM in rat plasma. Table S3: stabilities of the 13 constituents of RPM in rat plasma. [file 4508374.f1.pdf]

Table S1. Intra- and inter-day accuracy and precision for the determination of the 13 constituents of RPM in rat plasma

Comp.	Conc. （ng/mL）	Intra-day (n=6)			Inter-day (n=18)		
		Measured concentration （ng/mL）	Accuracy (%)	Precision (%)	Measured concentration （ng/mL）	Accuracy (%)	Precision (%)
Chrysophanol	1	0.98±0.075	97.7	7.72	0.94±0.083	93.6	8.88
	2	2.05±0.111	103	5.39	1.94±0.179	97.2	9.20
	27	28.3±2.04	105	7.19	23.5±0.938	87.1	3.99
	583	633±20.8	109	3.28	562±47.9	96.4	8.51
Emodin	0.1	0.11±0.007	108	6.92	0.11±0.006	106	6.09
	0.2	0.21±0.009	106	4.05	0.21±0.011	104	5.13
	2.7	2.84±0.014	105	6.67	2.85±0.267	106	9.36
	58.3	59.1±2.96	101	4.99	62.6±2.62	107	4.19
Aloe-emodin	0.1	0.10±0.007	96.8	6.78	0.09±0.007	92.6	7.66
	0.2	0.18±0.006	91.7	3.19	0.19±0.018	95.6	9.28
	2.7	2.55±0.133	94.4	5.21	2.43±0.142	90.0	5.83
	58.3	57.4±2.36	98.4	4.17	53.8±3.93	92.3	7.31
Rhein	0.5	0.53±0.036	107	6.78	0.49±0.035	97.8	7.13
	1	1.06±0.023	106	2.28	0.94±0.072	94.1	7.68
	13.5	14.5±0.532	107	3.68	13.0±0.709	96.1	5.47
	292	260±9.08	89.0	3.49	301±15.8	103	5.24
Physcion	0.5	0.50±0.038	99.2	7.67	0.51±0.037	101	7.33
	1	0.97±0.011	96.8	5.25	1.02±0.054	102	5.37
	13.5	13.8±0.44	102	3.22	14.1±1.11	105	8.23
	292	287±14.4	98.2	4.94	300±14.1	103	4.83

Questin	0.1	0.11±0.003	107	3.43	0.09±0.007	89.6	6.81
	0.2	0.21±0.008	103	4.15	0.18±0.016	92.4	7.94
	2.7	2.85±0.10	106	3.54	2.46±0.164	91.1	6.07
	58.3	52.0±1.75	89.2	3.03	52.7±2.25	90.5	3.86
Citreorosein	0.1	0.10±0.008	97.7	8.28	0.09±0.004	92.9	4.59
	0.2	0.19±0.014	93.7	7.48	0.19±0.008	93.4	4.39
	2.7	2.67±0.13	98.7	5.06	2.52±0.160	93.3	6.36
	58.3	56.8±3.87	97.5	6.82	59.5±1.62	102	2.73
Questinol	0.1	0.10±0.007	102	6.69	0.09±0.004	90.6	4.75
	0.2	0.20±0.010	102	4.70	0.19±0.010	94.4	5.07
	2.7	2.78±0.140	103	5.02	2.50±0.151	92.6	6.04
	58.3	57.0±2.01	97.8	3.61	54.0±3.13	92.6	5.80
TSG	0.3	0.30±0.024	101	7.89	0.31±0.015	102	5.04
	0.6	0.59±0.586	97.7	6.61	0.59±0.061	98.6	10.3
	8.1	7.36±0.283	90.9	3.85	7.75±0.762	95.7	9.84
	175	168.6±7.68	96.4	4.56	176±14.5	101	8.18
TG	0.1	0.10±0.007	97.2	7.38	0.09±0.007	93.3	7.74
	0.2	0.20±0.004	97.8	2.09	0.18±0.004	89.4	2.06
	2.7	2.68±0.146	99.1	5.46	2.55±0.171	94.4	6.72
	58.3	55.8±2.44	95.8	4.38	55.3±3.40	94.9	6.14
CG	0.1	0.10±0.005	102	4.59	0.10±0.002	98.1	1.83
	0.2	0.18±0.022	91.6	10.9	0.19±0.016	95.4	8.16
	2.7	2.46±0.101	91.1	3.74	2.42±0.113	89.8	4.18
	58.3	59.3±4.05	102	6.94	54.5±3.70	93.5	6.35

EG	0.1	0.10±0.007	95.4	7.37	0.09±0.006	90.7	6.75
	0.2	0.19±0.012	96.9	6.20	0.18±0.006	87.7	3.61
	2.7	2.75±0.114	102	4.16	2.56±0.170	94.7	6.66
	58.3	58.7±2.83	101	4.83	57.8±1.70	99.1	2.94
PG	0.1	0.11±0.003	107	3.20	0.09±0.009	94.8	9.26
	0.2	0.21±0.008	103	3.88	0.20±0.018	101	8.90
	2.7	2.86±0.098	106	3.42	2.38±0.141	88.2	5.91
	58.3	52.0±1.75	89.2	3.36	58.5±4.55	100	7.77

Table S2. Recovery and matrix effect of the 13 constituents of RPM in rat plasma

Compound	Nominal concentration (ng/mL)	Recovery (%, Mean ± SD)	Matrix effect	
			Absolute (%, Mean ± SD)	Relative (%, RSD)
Chrysophanol	2	96.2±4.25	93.3±11.2	12.0
	27	89.9±5.13	91.5±3.10	3.39
	583	96.5±6.71	89.5±2.69	3.00
Emodin	0.2	93.3±10.2	99.6±10.1	10.1
	2.7	102±6.91	104±9.42	9.02
	58.3	93.2±4.29	109±8.30	7.83
Aloe-emodin	0.2	98.7±7.22	104±11.1	10.6
	2.7	92.1±2.38	103±9.91	9.62
	58.3	94.6±2.39	98.9±7.43	7.51
Rhein	1	94.1±7.23	104±6.78	6.50
	13.5	96.1±5.25	102±11.7	11.5
	292	103±5.42	105±5.23	4.96
Physcion	1	97.3±4.63	98.3±10.8	11.0
	13.5	91.9±4.49	99.4±5.31	5.35
	292	93.4±4.50	98.5±0.78	0.79
Questin	0.2	102±5.02	108±9.87	9.17
	2.7	103±6.81	102±7.72	7.59
	58.3	106±7.52	103±7.53	7.31
Citreorosein	0.2	92.9±4.27	105±6.73	6.39
	2.7	91.2±1.87	101±8.19	8.13
	58.3	94.5±8.53	105±7.76	7.36
Questinol	0.2	90.6±4.30	104±5.81	5.59
	2.7	93.1±4.06	105±4.37	4.16
	58.3	91.5±7.25	94.2±4.05	4.30
TSG	0.6	103±5.14	58.0±7.58	13.1
	8.1	100±10.5	55.6±3.61	6.49
	175	104±6.09	59.4±6.73	11.3
TG	0.2	93.3±7.22	91.3±10.0	12.3
	2.7	94.6±4.02	88.0±5.27	6.00
	58.3	92.8±5.72	90.7±3.34	6.39
CG	0.2	107±3.31	75.1±3.91	5.21
	2.7	99.2±8.20	76.3±6.62	8.67
	58.3	97.0±3.74	79.5±10.7	13.4
EG	0.2	90.7±6.13	97.0±7.90	8.15
	2.7	94.2±2.47	93.4±4.70	5.03
	58.3	93.7±7.00	104±4.68	4.48
PG	0.2	94.8±8.77	68.9±5.16	8.76
	2.7	94.2±10.8	65.3±8.58	13.1
	58.3	95.4±5.23	72.9±7.72	10.6

Table S3. Stabilities of the 13 constituents of RPM in rat plasma

**Compound**	**Concentration （ng/mL）**	**Short-term**	**Post-preparative**	**Freeze–thaw (three cycles)**	**Long-term (–70 °C)**
Chrysophanol	2	98.4	98.9	102	93.3
	27	107	101	98.2	95.5
	583	104	107	104	99.3
Emodin	0.2	93.6	101	102	100
	2.7	105	103	104	102
	58.3	106	98.1	104	101
Aloe-emodin	0.2	105	96.4	99	103
	2.7	105	102	97.2	98.4
	58.3	104	92.6	101	102
Rhein	1	102	102	101	104
	13.5	107	103	106	103
	292	105	92.8	107	95.4
Physcion	1	102	104	98.0	96.7
	13.5	101	103	102	91.6
	292	100	98.4	101	94.1
Questin	0.2	104	94.2	90.9	92.4
	2.7	101	105	93.4	91.1
	58.3	106	92.5	93.7	90.5
Citreorosein	0.2	105	93.1	93.2	94.7
	2.7	104	103	101	100
	58.3	109	95.5	95.8	97.4
Questinol	0.2	107	93.1	94.7	94.3
	2.7	98.7	97.3	107	104
	58.3	102	90.4	93.7	92.9
TSG	0.6	103	106	101	106
	8.1	100	100	110	102
	175	98.9	102	104	103
TG	0.2	109	96.6	97.2	92.5
	2.7	92.3	108	107	102
	58.3	97	92.7	95.2	88.7
CG	0.2	102	98.1	99.1	91.6
	2.7	106	104	106	91.1
	58.3	96.9	103	96.0	102
EG	0.2	100	98.4	99.1	87.6
	2.7	103	107	106	101
	58.3	111	96.9	96.0	91.8
PG	0.2	112	94.8	97	95.9
	2.7	105	101	108	101

**Compound**	**Concentration（ng/mL）**	**Short-term**	**Post-preparative**	**Freeze–thaw (three cycles)**	**Long-term (–70 °C)**
	58.3	94.7	101	107	107
